# Effect of keratometric astigmatism on visual outcomes following small incision lenticule extraction

**DOI:** 10.3389/fmed.2022.982892

**Published:** 2022-10-18

**Authors:** Shengtao Liu, Lanhui Yu, Zhiyuan Lu, Chiwen Cheng, Xuejun Gu, Jingying Liu, Xingtao Zhou

**Affiliations:** ^1^Department of Ophthalmology and Optometry, Eye and ENT Hospital, Fudan University, Shanghai, China; ^2^NHC Key Laboratory of Myopia, Fudan University, Shanghai, China; ^3^Key Laboratory of Myopia, Chinese Academy of Medical Sciences, Shanghai, China; ^4^Shanghai Research Center of Ophthalmology and Optometry, Shanghai, China; ^5^Affiliated Eye Hospital of Nanchang University, Nanchang, China

**Keywords:** small incision lenticule extraction, high keratometric astigmatism, low keratometric astigmatism, myopia, visual outcomes

## Abstract

**Purpose:**

To investigate the influence of keratometric astigmatism on visual outcomes following small incision lenticule extraction (SMILE).

**Methods:**

Eighty eyes undergoing SMILE for myopia correction were classified into two groups based on preoperative keratometric astigmatism: low keratometric astigmatism (LA) and high keratometric astigmatism (HA) groups. Refractive outcomes, vector components, and changes in higher order aberrations (HOAs) were evaluated preoperatively and 6 months postoperatively.

**Results:**

At the postoperative 6-month visit, no significant difference was observed in the decentered distance between the HA and LA groups (HA: 0.17 ± 0.08 mm, LA: 0.16 ± 0.08 mm, *P* = 0.189). No significant differences in the correction index (*P* = 0.481), absolute angle of error (*P* = 0.104), or index of success (*P* = 0.147) were observed between the two groups. There was no significant difference in the induction of corneal aberrations between the two groups. Furthermore, there were no significant associations between the decentered distance and the vector components of astigmatic correction or induction of higher-order aberration in the HA group (*P* ≥ 0.294, *P* ≥ 0.112) or the LA group (*P* ≥ 0.323, *P* ≥ 0.080).

**Conclusions:**

SMILE for high keratometric astigmatism could achieve comparable treatment centration and visual quality to that of low keratometric astigmatism

## Introduction

Currently, small incision lenticule extraction (SMILE) is recommended for the treatment of myopic astigmatism up to 5.00 D (1). Different from laser in situ keratomileusis, with SMILE, the centration of treatment purely relies on the suction of the anterior cornea. Several studies have assessed the effect of treatment decentration on visual outcomes in SMILE, but little is known about the factors that could affect the achieved centration (2–5). General opinion has assumed that the morphology of the anterior cornea may affect lenticule decentration and visual outcomes (4, 5). Therefore, whether keratometric astigmatism, an important factor of anterior cornea, has an impact on visual outcomes needs to be further determined.

A previous study has attempted to investigate the visual outcomes between the high astigmatism eyes and the low astigmatism eyes (6). However, this previous study has classified the groups based on preoperative refractive astigmatism, not keratometric astigmatism. To the best our knowledge, there was no study investigating the visual outcomes following SMILE between different keratometric astigmatism, which may affect lenticule decentration in SMILE. Therefore, the current study aimed to compare optical zone decentration and visual outcomes following SMILE in eyes with low and high keratometric astigmatism.

## Materials and methods

### Collection of patients

In this prospective study, a total of eighty eyes of eighty patients who underwent SMILE for the correction of myopia and myopic astigmatism were recruited at the Affiliated Eye Hospital of Nanchang University (Nanchang, People's Republic of China) between October 2018 and May 2019. The inclusion criteria included age ≥ 18 y, the preoperative corrected distance visual acuity (CDVA) of 20/20 or better in all eyes and stable refraction for 2 y. Patients with systemic diseases, history of eye surgery or history of eye diseases were excluded. The target postoperative spherical equivalent (SE) was plano. Patients with keratometic astigmatism of lower than 2.0 D were included in low keratometric astigmatism (LA) group, and higher than 2.0 D were included in high keratometric astigmatism (HA) group. This prospective study was approved by the Ethical Committee of the Affiliated Eye Hospital of Nanchang University Review Board. All patients provided informed consent in accordance with the tenets of the Declaration of Helsinki.

### Surgical techniques

SMILE procedures were performed as described previously with the VisuMax femtosecond laser system (Carl Zeiss Meditec AG, Germany) (7). To eliminate the influence of cyclotorsion in astigmatism correction, we have compensated for it by rotating the patient's head (8). The patient was asked to fixate on a blinking target. After final confirmation that green light was coincident with the tear film center (tear film mark was concentric with the margin of the cone), suction was initiated. The intended thickness of the cap was set to 120 μm. The lenticule diameter was 6.2–7.0 mm and the cap diameter was 7.5 mm. The incision length and position were set at 2.0 mm and 90°. After laser treatment, the refractive lenticule was dissected and removed manually. All of the surgical procedures were performed by the same surgeon (SL).

### Measurement of optical zone decentration

A difference map of the tangential curvature was generated for each eye by using the preoperative and 6-month-postoperative Scheimpflug camera (Pentacam HR; Oculus, Wetzlar, Germany) exams. The method was introduced in our recently published study investigating optical zone centration accuracy (7). The optical zone was defined on the tangential topography difference map as the central zone up to the midperipheral power inflection point. The best-fitting circle and central grid were superimposed on the optical zone to determine the location of the optical zone center with reference to the corneal vertex (CV).

### Vector and aberration analysis

Vector analysis was performed for eyes with astigmatic correction based on the Alpins methods (9, 10). As suggested by Alpins, the target induced astigmatism (TIA), surgically induced astigmatism (SIA), difference vector (DV), magnitude of error (ME), angle of error (AE), correction index (CI) and index of success (IOS) were analyzed.

Corneal wavefront aberrations were measured before surgery and 6 months after surgery in a dark environment with a Scheimpflug camera (Pentacam HR; Oculus, Wetzlar, Germany). The coefficients were analyzed for a standardized diameter of 6.0 mm. The root mean square (RMS) values of the coma and total higher-order aberrations (HOAs) were calculated. The coefficients of vertical coma, horizontal coma, and spherical aberration were analyzed because they are clinically significant in visual quality (2, 7). All measurements were performed by a single experienced operator with double blind method. Additionally, all measurements were repeated 3 times.

### Corneal densitometry analysis

Corneal Densitometry (CD) was quantified with the Pentacam HR. The data are displayed on the CD map, which is divided into three anatomical layers based on the depth: the anterior layer (120 μm anteriorly), posterior layer (60 μm posteriorly), and central layer (at mid-distance between the two layers). In addition, four concentric radial zones are defined around the corneal apex (0–2, 2–6, 6–10, and 10–12 mm). Values at the outermost zone of 10–12 mm have the weakest reliability and reproducibility and were excluded.

### Statistical analyses

All statistical analyses were performed using IBM SPSS Statistics 24.0 (SPSS Inc, Chicago, IL). Mean ± SD was used for quantitative variables. Differences were considered statistically significant when the *P* values were <0.05. The Kolmogorov-Smirnov test was used to confirm data normality. Independent *t*-tests were used to compare clinical variables, decentered displacement, astigmatic vector components and induced corneal HOAs between the two groups. Pearson analyses were used to determine the associations.

## Results

### Clinical characteristics of the enrolled patients

A total of 40 eyes were included in the HA group (mean keratometric astigmatism: 2.72 ± 0.24 D, range: 2.50–3.40 D) and 40 eyes in the LA group (mean keratometric astigmatism: 0.82 ± 0.36 D, range: 0.20–1.50 D). Demographic data are presented in Table 1.

**Table 1:** Clinical characteristics of eyes that underwent SMILE.

**Characteristic**	**LA Group**	**HA Group**	***P***
Patients (eyes, n)	40, 40	40, 40	–
Age (y)	23.8 ± 4.4 (18 to 35)	23.0 ± 5.0 (18 to 44)	0.226
Sex (% women)	60%	55%	-
**Refractive errors (D)**			
Spherical	– 4.64 ± 1.61 (– 2.00 to – 8.50)	– 4.51 ± 1.67 (– 0.50 to – 7.25)	0.715
Cylindrical	– 0.68 ± 0.25 (– 0.25 to – 1.25)	– 2.03 ± 0.60 (– 1.00 to – 3.25)	<0.001^*^
MRSE	– 5.00 ± 1.58 (– 2.25 to – 8.75)	– 5.52 ± 1.65 (– 1.75 to – 8.63)	0.141
Optical zone (mm)	6.54 ± 0.10 (6.50 to 7.00)	6.54 ± 0.16 (6.20 to 7.00)	0.665
**Keratometry (D)**			
Flat keratometry	42.35 ± 1.37 (40.1 to 45.3)	42.00 ± 1.25 (39.5 to 45.4)	0.254
Steep keratometry	43.19 ± 1.42 (40.8 to 46.1)	44.43 ± 1.43 (42.0 to 48.1)	<0.001^*^
Average keratometry	42.78 ± 1.38 (40.50 to 45.60)	43.23 ± 1.26 (40.70 to 46.60)	0.127
Keratometric astigmatism	0.82 ± 0.36 (0.20 to 1.50)	2.72 ± 0.24 (2.50 to 3.40)	<0.001^*^

LA, low astigmatism; HA, high astigmatism; D, diopters; MRSE, manifest refraction spherical equivalent. Values presented as means ± standard deviation (range).

* Significant difference between the LA and HA groups (*t* test).

### Refractive outcomes

**Figure 1:**
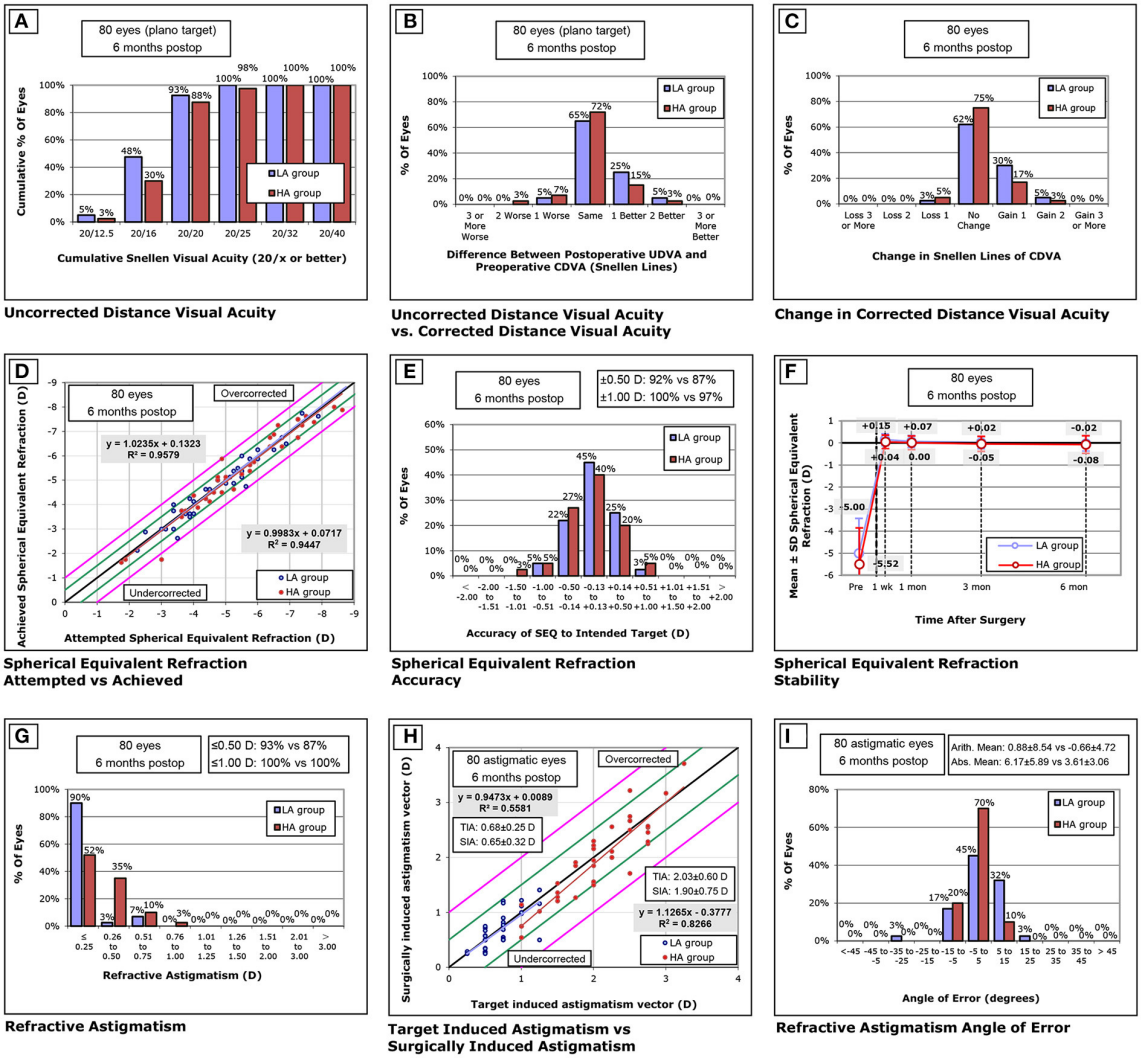
Visual outcomes at 6 months after SMILE. **(A)** uncorrected distance visual acuity (UDVA) outcomes. **(B)** postoperative UDVA and preoperative corrected distance visual acuity (CDVA). **(C)** change in CDVA. **(D)** distribution of achieved spherical equivalent outcomes. **(E)** spherical equivalent refractive accuracy. **(F)** stability of spherical equivalent refraction. **(G)** refractive astigmatism. **(H)** target induced vs. surgically induced astigmatism vectors, and **(I)** refractive astigmatism angle of error distribution at 6 months postoperatively. D, diopters.

At the postoperative 6-month visit, 88.0% (35/40) of treated eyes for the HA group and 93.0% (37/40) of treated eyes for the LA group achieved a uncorrected distance visual acuity (UDVA) of 20/20 or better (Figure 1A). Relative to the preoperative CDVA, 18% (7/40) and 30% (12/40) of treated eyes in the HA and LA groups, respectively, exhibited a gain of one or more lines in the postoperative UDVA (Figure 1B). Similarly, 95.0% (38/40) of treated eyes for the HA group and 97.0% (39/40) of treated eyes for the LA group exhibited unchanged or better CDVA (Figure 1C). A scatter plot of the attempted vs. the achieved SE correction is presented in Figure 1D. After surgery, the SE in 87.0% (35/40) of treated eyes for the HA group and 92% (37/40) of treated eyes for the LA group were within ±0.50 D (Figure 1E). The change in the manifest SE is shown in Figure 1F. As for astigmatism correction, 87.0% (35/40) of treated eyes for the HA group and 93% (37/40) of treated eyes for the LA group had postoperative astigmatism within 0.50 DC (Figure 1G). Scatterplots of the TIA vs. SIA vectors and the distribution of AE are shown in Figures 1H,I, respectively.

### Optical zone decentration

**Figure 2:**
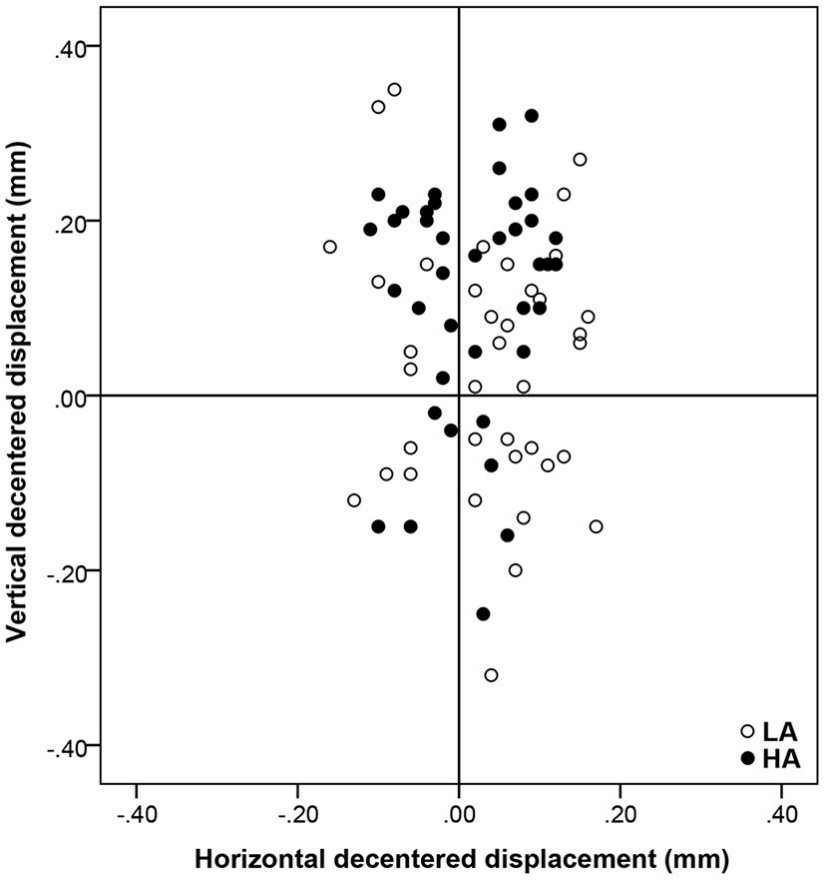
The location of optical zone center and the distribution of decentration distance relative to the corneal vertex. Positive vertical coordinates stand for superior displacements and negative for inferior ones. Positive horizontal coordinates stand for nasal displacements and negative for temporal ones.

The mean total decentration was 0.17 ± 0.08 mm (range: 0.03–0.33 mm) and 0.16 ± 0.08 mm (range: 0.02–0.36 mm) for the HA group and LA group, respectively, and there was no significant difference between the two groups (*P* = 0.189). From the distributions in Figure 2, the locations of the optical zone center in the LA group and HA group tended to be slight superior on average.

### Vector analysis

The preoperative cylindrical errors were 2.03 ± 0.60 D for the HA group and 0.68 ± 0.25 D for the LA group. There were no significant differences in the CI (*P* = 0.481), absolute AE (*P* = 0.104), or IOS (*P* = 0.147) between the two groups. The DV, defined as the postoperative residual astigmatism, was significantly higher in the HA group than that in the LA group (*P* < 0.001). No significant association was found between total decentration and DV, ME, IOS, CI, or absolute AE (*r* = 0.115, *P* = 0.480 for HA; *r* = 0.128, *P* = 0.429 for LA) (Table 2).

**Table 2:** Vector analysis results of astigmatism correction at 6 months after SMILE.

	**LA Group**			**HA Group**			***P*** **value**
	**Mean ±SD**	**Correlation with decentration distance**	***P*** **value for correlation**	**Mean ±SD**	**Correlation with decentration distance**	***P*** **value for correlation**	
TIA (D)	0.68 ± 0.25	–	–	2.03 ± 0.60	–	–	<0.001^*^
SIA (D)	0.65 ± 0.32	–	–	1.90 ± 0.75	–	–	<0.001^*^
DV (D)	0.20 ± 0.18	– 0.026	0.872	0.40 ± 0.20	0.170	0.294	<0.001^*^
ME (D)	– 0.03 ± 0.21	– 0.037	0.823	– 0.12 ± 0.32	0.023	0.890	0.084
AE (degree)	0.88 ± 8.54	– 0.160	0.323	– 0.66 ± 4.72	0.071	0.665	0.321
Absolute AE (degree)	6.17 ± 5.89	0.128	0.429	3.61 ± 3.06	0.115	0.480	0.104
CI	0.96 ± 0.30	−0.030	0.856	0.92 ± 0.17	−0.058	0.722	0.481
IOS	0.31 ± 0.27	0.044	0.788	0.21 ± 0.11	0.122	0.452	0.147

LA, low astigmatism; HA, high astigmatism; TIA, target induced astigmatism; SIA, surgically induced astigmatism; DV, dufference vector; ME, magnitude of error; AE, angle of error; CI, correction index; IOS, index of success. Values presented as means ± standard deviation.

* Significant difference between the LA and HA groups (*t* test).

### Wavefront aberration analysis

At 6 months postoperatively, induced changes in total HOAs (*P* = 0.323), RMS coma (*P* = 0.817), vertical coma (*P* = 0.301), horizontal coma (*P* = 0.362), and spherical aberration (*P* = 0.697) showed no significant difference between the HA group and the LA group. There were no significant relationship between the magnitudes of total decentration and induced corneal aberrations in the HA group (*P* = 0.136 for total HOAs; *P* = 0.316 for RMS coma; *P* = 0.855 for spherical aberration; *P* = 0.681 for vertical coma; and *P* = 0.112 for horizontal coma) or the LA group (*P* = 0.080 for total HOAs; *P* = 0.228 for RMS coma; *P* = 0.735 for spherical aberration; *P* = 0.113 for vertical coma; and *P* = 0.440 for horizontal coma) (Table 3).

**Table 3:** Changes in corneal aberrations at 6 months after SMILE.

	**LA Group**			**HA Group**			***P*** **value**
	**Mean ±SD**	**Correlation with decentration distance**	***P*** **value for correlation**	**Mean ±SD**	**Correlation with decentration distance**	***P*** **value for correlation**	
Horizontal coma	0.05 ± 0.12	0.125	0.440	0.07 ± 0.12	– 0.255	0.112	0.362
Vertical coma	– 0.12± 0.11	– 0.255	0.113	– 0.14 ± 0.16	0.067	0.681	0.301
RMS coma	0.03± 0.10	0.195	0.228	0.06 ± 0.17	0.163	0.316	0.817
Spherical aberration	0.06± 0.10	0.055	0.735	0.06 ± 0.11	0.030	0.855	0.697
HOAs	0.14± 0.11	0.280	0.080	0.17 ± 0.16	0.240	0.136	0.323

LA, low astigmatism; HA, high astigmatism; RMS, root mean square; HOAs, higher-order aberrations. Values presented as means ± standard deviation.

### Corneal densitometry analysis

At postoperative 6 months, a slight increase in CD at anterior 0–6 mm was observed in both groups. Additionally, no significant change in CD relative to baseline was observed at central and posterior layer in both groups. The change in CD was similar between groups at postoperative 6 months in the corresponding corneal zones (all *P* ≥ 0.060) (Table 4).

**Table 4:** Changes in corneal densitometry at 6 months after SMILE.

	**Anterior layer**			**Central layer**			**Posterior layer**			**Total**		
	**0–2 mm**	**2–6 mm**	**6–10 mm**	**0–2 mm**	**2–6 mm**	**6–10 mm**	**0–2 mm**	**2–6 mm**	**6–10 mm**	**0–2 mm**	**2–6 mm**	**6–10 mm**
LA Group	0.82 ± 2.59	1.15 ± 2.29	– 0.45 ± 2.71	– 0.01 ± 1.96	0.43 ± 1.82	– 0.18 ± 2.25	– 0.82 ± 1.31	– 0.05 ± 1.29	– 0.15 ± 1.96	0.01 ± 1.80	0.53 ± 1.67	– 0.26 ± 2.21
LA Group	0.58 ± 1.61	1.15 ± 1.80	– 0.12 ± 2.20	0.15 ± 1.64	0.50 ± 1.74	0.44 ± 1.86	– 0.54 ± 0.84	0.16 ± 1.02	0.57 ± 1.31	0.07 ± 1.25	0.61 ± 1.46	0.25 ± 1.75
*P*	0.866	0.908	0.554	0.338	0.802	0.185	0.086	0.421	0.060	0.500	0.613	0.261

LA, low astigmatism; HA, high astigmatism; Values presented as means ± standard deviation.

## Discussion

In our study, most treated eyes achieved an uncorrected distance visual acuity of 20/20 or better and showed unchanged or better corrected distance visual acuity. These results indicate that SMILE was safely and effectively performed in the eyes with both LA and HA. A greater proportion of LA eyes (92%) achieved spherical equivalent within ± 0.50 D compared to HA eyes (87.0%). Furthermore, the postoperative residual astigmatism was significantly higher in the HA group than in the LA group, a result similar to those of previous studies reported (6, 11). Although SMILE shows high predictability, there has been a tendency toward undercorrection when treating high astigmatism.

In the current study, there was no significant difference in decentration distances between the HA group and LA groupt (*P* = 0.189). We attributed the comparable optical zone decentration to the comparable tear film mark (touch zone), which guiding lenticule centration as we described previously (7). We further clarified that keratometric astigmatism did not affect the tear film mark decentration, because the asymmetry of curvature would be eliminated with the aggravated corneal compression (12).

Previous studies have suggested that decentration mainly affects the induction of HOA, but not the astigmatic vector results in SMILE (5, 6). Our results also showed no association between decentration distance and astigmatic vector results in either group. Therefore, we could conclude that mild decentration after SMILE was insufficient to affect astigmatism correction, even for patients with high keratometric astigmatism. Although no significant difference was observed in astigmatic vector results between the LA and HA groups, there seems to be a trend toward better treatment alignment with higher keratometric astigmatism. The distribution in AE, shown in Figure 1I, also suggested more treated eyes in the HA group within 5°. We suspected that lower absolute AE could be attributable to better evaluation of axis location in high keratometric astigmatism (11, 13). Previous studies have also suggested that wider differences in axis location tended to exist in eyes with low astigmatism (14, 15).

In the current study, there was no significant difference in the induction of corneal aberrations between the HA and LA groups. Jun et al. also found that induced HOA and coma in high astigmatism were comparable to that of moderate astigmatism (16). Huang et al. demonstrated that the induced coma and SA were greater in eyes with greater decentration in the HA group, but not in the LA group (6). They suspected that a decentration of > 0.20 mm in eyes with HA would result in greater sensitivity between induction of coma and decentration distance after SMILE. Lee et al. also observed a similar significant change in aberrations when decentration distance exceeded 0.335 mm (17). Due to the mean total decentrations of both the groups within the tolerance range, no significant association was found between decentration distance and induced aberrations in the present study. Ding et al. also demonstrated that the induced aberrations were not related to optical zone decentration in either group (18). Therefore, accurate centration could compensate for the phenomenon that HA amplifies the effect of optical zone decentration on corneal aberrations. Additionally, we also notice that the change in CD was similar between groups at postoperative 6 months in the corresponding corneal zones.

The limitations of this study include its relatively small sample size and lack of FOZ measurement. Thus, in further studies, more investigations of visual outcomes with FOZ for patients with astigmatism are warranted. In summary, our data confirmed that SMILE for high keratometric astigmatism could achieve comparable treatment centration and visual quality to that of low keratometric astigmatism.

## Data availability statement

The raw data supporting the conclusions of this article will be made available by the authors, without undue reservation.

## Ethics statement

This prospective study was approved by the Ethical Committee of the Affiliated Eye Hospital of Nanchang University Review Board. All patients provided informed consent in accordance with the tenets of the Declaration of Helsinki. The patients/participants provided their written informed consent to participate in this study.

## Author contributions

SL conceived and designed the study. SL, LY, ZL, CC, XG, and JL analyzed the data and wrote the manuscript. LY, ZL, CC, and XG obtained the samples and clinical records. SL, JL, and XZ reviewed and revised the manuscript. XZ supervised the study. All authors contributed to the article and approved the final version of the manuscript.

## Funding

This work was supported by National Natural Science Foundation of China (No. 81770955) and Joint research project of new frontier technology in municipal hospitals (SHDC12018103).

## Conflict of interest

The authors declare that the research was conducted in the absence of any commercial or financial relationships that could be construed as a potential conflict of interest.

## Publisher's note

All claims expressed in this article are solely those of the authors and do not necessarily represent those of their affiliated organizations, or those of the publisher, the editors and the reviewers. Any product that may be evaluated in this article, or claim that may be made by its manufacturer, is not guaranteed or endorsed by the publisher.
